# Glucose‐dependent insulinotropic polypeptide receptor antagonist treatment causes a reduction in weight gain in ovariectomised high fat diet‐fed mice

**DOI:** 10.1111/bph.15894

**Published:** 2022-07-06

**Authors:** Geke Aline Boer, Jenna Elizabeth Hunt, Maria Buur Nordskov Gabe, Johanne Agerlin Windeløv, Alexander Hovard Sparre‐Ulrich, Bolette Hartmann, Jens Juul Holst, Mette Marie Rosenkilde

**Affiliations:** ^1^ Novo Nordisk Foundation Center for Basic Metabolic Research, Faculty of Health and Medical Sciences University of Copenhagen Copenhagen Denmark; ^2^ Department of Biomedical Sciences, Faculty of Health and Medical Sciences University of Copenhagen Copenhagen Denmark; ^3^ Antag Therapeutics ApS Copenhagen Denmark

**Keywords:** antagonist, GIP receptor, glucose‐dependent insulinotropic polypeptide (GIP), obesity, pharmacokinetics

## Abstract

**Background and purpose:**

The incretin hormone, gastric inhibitory peptide/glucose‐dependent insulinotropic polypeptide (GIP), secreted by the enteroendocrine K‐cells in the proximal intestine, may regulate lipid metabolism and adiposity, but its exact role in these processes is unclear.

**Experimental approach:**

We characterized *in vitro* and *in vivo* antagonistic properties of a novel GIP analogue, mGIPAnt‐1. We further assessed the *in vivo* pharmacokinetic profile of this antagonist, as well as its ability to affect high‐fat diet (HFD)‐induced body weight gain in ovariectomised mice during an 8‐week treatment period.

**Key results:**

mGIPAnt‐1 showed competitive antagonistic properties to the GIP receptor *in vitro* as it inhibited GIP‐induced cAMP accumulation in COS‐7 cells. Furthermore, mGIPAnt‐1 was capable of inhibiting GIP‐induced glucoregulatory and insulinotropic effects in vivo and has a favourable pharmacokinetic profile with a half‐life of 7.2 h in C57Bl6 female mice. Finally, sub‐chronic treatment with mGIPAnt‐1 in ovariectomised HFD mice resulted in a reduction of body weight and fat mass.

**Conclusion and Implications:**

mGIPAnt‐1 successfully inhibited acute GIP‐induced effects *in vitro* and *in vivo* and sub‐chronically induces resistance to HFD‐induced weight gain in ovariectomised mice. Our results support the development of GIP antagonists for the therapy of obesity.

**Abbreviations**
HFD: high‐fat diet
val‐pyr: valine pyrrolidide
WAT: white adipose tissue

**What is already known?**

- GIP may regulate lipid metabolism and adiposity but its role in these processes is unclear.

**What does this study add?**

- We characterized *in vitro* and *in vivo* antagonistic properties of a novel GIP analogue, mGIPAnt‐1.
- Sub‐chronic treatment with mGIPAnt‐1 in ovariectomised HFD mice reduced body weight and fat mass.

**What is the clinical significance?**

- We have demonstrated a novel tool to study the effects of GIP antagonism in obesity.
- Our results support the development of GIP antagonists for the therapy of obesity.

## 1 INTRODUCTION

Following ingestion of a meal, the enteroendocrine K‐cells in the proximal gut secrete the 42‐amino acid hormone, gastric inhibitory polypeptide/glucose‐dependent insulinotropic polypeptide (GIP) (Buffa et al., 1975; Jörnvall et al., 1981). GIP is an incretin hormone that stimulates insulin production through activation of its receptor (GIP receptor) on pancreatic β‐cells (McIntyre et al., 1964). Additionally, this receptor is expressed in various other tissues, including adipose tissue, bone, the central nervous system (CNS) and the heart, suggesting that GIP has other functions in the body (Adriaenssens et al., 2019; Bollag et al., 2000; Usdin et al., 1993; Yip et al., 1998). Research has been especially directed towards elucidating the effect of GIP on adipose tissue and its role in the pathogenesis of obesity.

Besides GIP receptor expression on adipocytes (Yip et al., 1998), other indications for a link between GIP and obesity include the findings of increased circulating GIP levels in both obese individuals (Calanna et al., 2013) and subjects given a high‐fat diet (HFD) (Brøns et al., 2009). *In vitro* studies have shown that GIP increases fatty acid uptake in adipocytes (Kim et al., 2007; Yip et al., 1998) and in humans, where infusion of GIP during a high‐insulin, high‐glucose clamp increased triglyceride deposition in subcutaneous adipose tissue (Asmar et al., 2010). Furthermore, GIP receptor knockout (GIP receptor KO, GIPR^−/−^) mice are resistant to diet‐induced obesity (Miyawaki et al., 2002) and transgenic rescue of the GIP receptor in adipose tissue in the same mouse model counteracted this resistance (Ugleholdt et al., 2011). Interestingly, double incretin receptor knockout (DIRKO) and glucagon‐like peptide‐1 (GLP‐1) receptor KO mice (Glp1r^‐/‐^) also showed resistance to diet‐induced obesity, despite a significant increase in food intake in the GLP‐1 receptor KO mice (Drucker, 2003; Hansotia, 2004; Hansotia et al., 2007).

Following these findings, considerable effort has been exerted towards the development of antagonists of the GIP receptor for the treatment of obesity. Both GIP and GIP receptor neutralizing antibodies and treatment with SKL‐14959, a GIP antagonistic small‐molecule compound a GIP antagonist, induced resistance to weight gain in HFD‐fed mice (Boylan et al., 2015; E. A. Killion et al., 2018; Nakamura et al., 2018). Furthermore, active immunization against GIP has, in preclinical studies, shown to induce weight loss in obese mice (Fulurija et al., 2008), although not all studies could show this effect (Irwin et al., 2012). Peptide analogues based on a naturally occurring C‐ and N‐terminally truncated GIP variant, GIP(3‐30)NH_2_ , were found to antagonize the GIP receptor and reduced obesity and improved metabolic control in mice (Hansen et al., 2016; Pathak et al., 2015). The most potent antagonist of the truncated GIP variants, GIP(3‐30)NH_2_ was shown to inhibit GIP receptor signalling in human adipocytes (Gabe et al., 2018) and to inhibit triglyceride deposition in subcutaneous adipose tissue in humans during *in vivo* infusions (Asmar et al., 2017). Interestingly, treatment with GIP agonists has also been shown to reduce body weight or weight gain in preclinical studies; both when given alone and in combination with an agonist of the GLP‐1 receptor (Mroz et al., 2019; Nørregaard et al., 2018). An explanation for this could be that chronic treatment with a GIP agonist results in desensitization of the GIP receptor, resulting in functional antagonism of the receptor (Elizabeth A. Killion et al., 2020). However, recent studies have shown that even following internalization after GIP binding to its receptor, the internalized complexes retain their ability to continue cAMP signalling and further downstream signalling (Holst, 2021).

In this study, we have characterized the *in vitro* antagonistic properties of a novel GIP analogue that was optimized for chronic use in murine studies, hereafter indicated as mGIPAnt‐1. We also assessed the pharmacokinetic profile of this antagonist, as well as its ability to acutely inhibit GIP‐induced glucoregulatory actions *in vivo*. Finally, we studied how sub‐chronic treatment with mGIPAnt‐1 affected HFD‐induced body weight gain in ovariectomised mice. The ovariectomised model resembles the post‐menopausal state and the oestrogen deficiency‐induced in this model results in increased body weight, abdominal fat mass and insulin resistance compared to sham‐operated mice (McElroy & Wade, 1987). GIP receptor KO mice are resistant to weight gain (Isken et al., 2008) induced by ovariectomy. With this study, we also used the ovariectomised mouse model to assess whether our antagonist was able to induce similar beneficial effects.

## 2 METHODS

### 2.1 *In vitro* studies

COS‐7 cells were cultured at 10% CO_2_ and 37°C in Dulbecco's Modified Eagle Medium 1885 supplemented with 10% foetal bovine serum, 2‐mM glutamine, 180‐units ml^−1^ penicillin and 45‐g·ml^−1^ streptomycin. Transient transfection of the cells with either human or mouse GIP receptor was performed using the calcium phosphate precipitation method as previously described (van der Velden et al., 2021). A second set of cells was transiently transfected with either mouse GLP‐1 or GLP‐2 receptor. Following the day after transfection, the cells were seeded in white 96‐well plates with a density of 35.000 cells per well. The next day the assay was initiated by washing the cells with HEPES buffered saline (HBS) followed by an incubation step with HBS and 1‐mM 3‐isobutyl‐1‐methylxanthine for 30 min at 37°C. For agonist studies, the ligands were then added and incubated for an additional 30 min at 37°C. To test for antagonistic properties, the cells were first preincubated for 10 min with the antagonist with subsequent addition of the agonist and incubated for an additional 20 min. The HitHunter® cAMP assay (Eurofins DiscoverX, Fremont, USA) was carried out according to the manufacturer's protocol. Luminescence was measured by PerkinElmer™ Envision 2014 Multilabel Reader.

### 2.2 Animal studies

Female C57Bl/6Jrj mice (8 weeks old) were obtained from Janvier Labs (Saint Berthevin Cedex, France) and allowed to acclimatize prior to procedures in groups of 4 for 1 week in individually ventilated cages with a 12‐h light cycle with *ad libitum* access to standard chow and water. Animals were randomly assigned to the different equally sized treatment groups and the investigators were blinded regarding treatment. Animal studies are reported in compliance with the ARRIVE guidelines (Percie du Sert et al., 2020) and with the recommendations made by the *British Journal of Pharmacology* (Lilley et al., 2020). Animal experiments were performed with permission from the Danish Animal Experiments Inspectorate (licence 2013‐15‐2934‐00833) and the local ethical committee following the guidelines of Danish legislation governing animal experimentation (1987) and the National Institutes of Health (publication number 85‐23). All efforts were made to diminish animal sufferings and animal numbers used.

#### 2.2.1 Pharmacokinetic analysis

To assess mGIPAnt‐1 half‐life following subcutaneous (s.c.) administration, mice (*n* = 6) received a single s.c. administration of 300‐nmol·kg^−1^ mGIPAnt‐1. Retro‐orbital blood samples (50 μl) were taken using EDTA coated glass capillaries at (1) *t* = 0, 0.5, 1, 1.5, 2 and 2.5 h, (2) *t* = 2.5, 3, 4, 6, 8 and 22 h, or (3) *t* = 22, 24, 26, 28, 30 and 32 h and immediately added to Eppendorf tubes (Eppendorf, Hamburg, Germany) and centrifuged at 3500*g*, 20 min, 4°C. Plasma was transferred into Eppendorf tubes on dry ice and stored at −20°C for analysis.

#### 2.2.2 *In vivo* biological activity of mGIPAnt‐1

To assess the biological activity of mGIPAnt‐1, we performed an intraperitoneal glucose tolerance test. Mice were fasted for 4 h and given either mGIPAnt‐1 (25‐μmol·kg^−1^; s.c.) or vehicle at *t* = −90 min. Group sizes were as follows:‐ Vehicle + Vehicle, *n* = 10, Vehicle + GIP, *n* = 12 and mGIPAnt‐1 + GIP, *n* = 8. Varying group numbers were due to unsuccessful administration of one of the four compounds as confirmed by measurements of plasma concentrations. At *t* = −10 all mice were given an intraperitoneal (i.p.) injection with dipeptidyl peptidase‐4 (DPP‐4) inhibitor valine pyrrolidide followed by s.c. injection of either 25 nmol·kg^−1^ GIP (Synthetic mouse GIP(1‐42) or vehicle. At *t* = 0 min, mice received an i.p. injection with 0.5‐g·kg^−1^ glucose. Blood glucose was measured from the tail vein using a hand‐held glucometer (Accu‐Chek Mobile, Roche, Basel, Switzerland) at *t* = 0, 10, 30, 45, 60, 120 and 180 min. Retro‐orbital blood samples (50 μl) were taken using EDTA coated glass capillaries (Vitrex, Vasekær, Denmark) at *t* = 0, 15, 30 and 60, 120 and 180 min and centrifuged at 3500*g*, 20 min, 4°C. Plasma was transferred into Eppendorf tubes on dry ice and stored at −20°C for later insulin measurements.

#### 2.2.3 Ovariectomy

Prior to bilateral ovariectomy surgery and once daily for 48 h following, mice received analgesics through s.c. injection of carprofen (Rimadyl; 5 mg·kg^−1^). Anaesthesia was induced in an induction chamber with 5% isoflurane and maintained with 2% isoflurane through a nose cone during the procedure. Proper respiration was ensured with a flow of oxygen through the nose cone and body temperature was maintained at 37°C by placing the mice on a heating pad. Mice were placed in a prone position on a sterile surgery pad and the area of incision was shaved and sterilized with 70% ethanol. Two dorsal‐lateral incisions were made and the ovary and attached fat pad were individually pulled out of the abdominal cavity. The fallopian tubes were ligated and the ovaries were dissected, then the fat pad was carefully placed back in the abdominal cavity. The skin incision was closed using wound clips (Reflex, Alzet, Cupertino, CA, USA). In half of the animals, a microtransponder (Datamars, Lamone, Switzerland) was inserted and animals were placed in an HM‐2 cage with the intention to measure food intake (MBrose, Faaborg, Denmark) and left to recover and acclimatize for a week. The remaining animals were placed back in their home cages to recover for a week. The division of mice into HM‐2 cages and home cages was randomized. Uterine atrophy was confirmed in all mice upon termination of the study, indicating successful ovariectomy.

#### 2.2.4 HFD studies

A week after ovariectomy, at 10 weeks of age, animals were randomized into groups and placed on a 60 kcal% HFD (HFD, D12492, Research Diets, New Brunswick, NJ, USA) or a control diet (10 kcal% fat, D12450J, Research Diets) for 8 weeks. Group sizes were as follows: Vehicle/control diet, *n* = 12; mGIPAnt‐1/control diet, *n* = 12; Vehicle/HFD, *n* = 11; mGIPAnt‐1/HFD, *n* = 12. The reduction of animal numbers in group Vehicle/HFD was due to unexpected loss of an animal. Animals received a daily s.c. injection at 4:30 PM with mGIPAnt‐1 (300‐nmol·kg^−1^) or vehicle. Body weight and body composition were assessed weekly (LF90II body composition analyzer, Bruker, Billerica, MA, USA). On the final experimental day, animals were anaesthetized with an intraperitoneal injection of 90 mg·kg^−1^ ketamine and 10‐mg·kg^−1^ xylazine. The microchip was quickly removed from HM‐2 cage animals and a final body composition measurement was performed on all animals. A terminal retro‐orbital blood sample was taken, mice were killed through cervical dislocation and tissues were quickly dissected and weighed.

#### 2.2.5 Glucose tolerance test

After 7 weeks HFD and mGIPAnt‐1 treatment, an oral glucose tolerance test was performed. Animals were fasted from 08:00 AM to 11:00 AM and received 2‐g·kg^−1^ glucose through oral gavage. Blood glucose was measured in the tail vein samples using a hand‐held glucometer (Accu‐Chek Mobile, Roche) at *t* = 0, 15, 30, 45, 60, 120 and 180 min. Retro‐orbital blood samples (75 μl) were taken using EDTA coated glass capillaries at *t* = 0, 15, 30 and 60 min and centrifuged at 3500*g*, 20 min, 4°C. Plasma was transferred into Eppendorf tubes on dry ice and stored at −20°C for later analysis of insulin measurements.

### 2.3 Biochemical analysis

#### 2.3.1 Radioimmunoassay

mGIPAnt‐1 levels were measured by an in‐house radioimmunoassay using a polyclonal antibody recognizing a mid‐regional part of GIP (code no. 95234) (Gasbjerg et al., 2017). mGIPAnt‐1 was used as standard and the assay buffer was a 80‐mM phosphate buffer pH 7.5, containing (in final concentrations) 0.1% HSA, 10‐mM EDTA, 500‐KIU ml^−1^ aprotinin and 0.01‐mM valine pyrrolidide. Samples were carefully diluted to ensure that samples would fall within the measurable range of the assay. The tracer was ^125^I‐monolabelled human GIP(1‐42).

#### 2.3.2 ELISA

Insulin levels in intraperitoneal glucose tolerance test and oral glucose tolerance test plasma samples were measured using a mouse insulin ELISA (catalogue no. 10‐1247‐10; Mercodia AB, Uppsala, Sweden). Adiponectin levels in terminal plasma samples were measured using a mouse adiponectin ELISA kit (catalogue no. 80569, Crystal Chem, Elk Grove Village, IL, USA). Adiponectin samples were carefully diluted in kit‐specific assay buffer. The manufacturer's instructions were followed closely.

##### Triglyceride and glycerol

Triglyceride and free glycerol levels in terminal plasma samples were determined using a triglyceride and free glycerol kit (TR0100‐1KT, Sigma‐Aldrich, St. Louis, MO, USA).

### 2.4 Data and statistical analysis

The data and statistical analysis comply with the recommendations of the *British Journal of Pharmacology* on experimental design and analysis in pharmacology (Curtis et al., 2018). Studies were designed to generate groups of equal size and any variation in group size within an experiment is due to either unsuccessful administration of an agent as confirmed by measurements of plasma concentrations or through unexpected loss of an animal. The pharmacokinetic studies involved six animals. *In vivo* biological activity of mGIPAnt‐1 was determined with an intraperitoneal glucose tolerance test with *n* = 8–12 and the HFD study was performed with *n* = 11–12, ), where *n* = number of independent values. Sample sizes for experiments were determined based on previous studies (Boer, Hartmann, & Holst, 2021; Boer, Keenan, et al., 2021). To ensure blinding regarding treatment, compounds were coded by an independent party.

**FIGURE 1:**
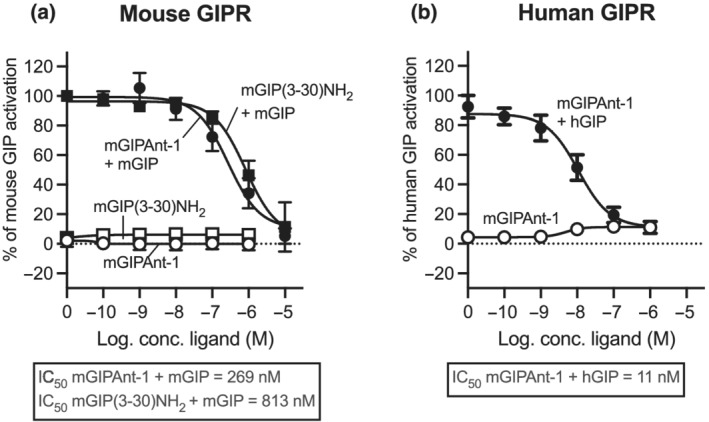
*In vitro* signalling profile of mGIPAnt‐1 on the mouse and human glucose‐dependent insulinotropic polypeptide receptor/(GIPR). COS‐7 cells were transiently transfected with (a) mouse GIPR or (b) human GIPR and assayed for cAMP accumulation. To test for agonism, increasing concentration of the ligand was added. To test for antagonism, increasing concentrations of the antagonist were added together with a fixed concentration of either mouse GIP(1‐42) or human GIP(1‐42) corresponding to 50%–80% of E_max_ on the mouse GIP or human GIP receptor, respectively. Data are shown as mean ± SE from *n* = 5 independent experiments

To facilitate comparisons of assay outputs (cAMP assays), the results (of duplicate measurements) were normalized, with the response to 10 pM of GIP(1‐42) set as 100%. All ligand concentrations are reported as log‐transformed. The IC_50_ log values can be found in Figure 1. In Figure 3c, we have subtracted glucose levels from baseline to show the net differences in glucose levels in our different treatment groups. The raw data is shown in Figure 3b.

Half‐life (t½) of mGIPAnt‐1 administered s.c. was, determined from the concentration versus time curves after semi‐logarithmical transformation and calculated with the following formulas: *k* = (ln (C_t1_) − ln (C_t2_))/𝛥t and t½ = ln(2)/k with C_t1_ at *t* = 1.5 h and C_t2_ at *t* = 32 h. Results are presented as mean ± SE. Data were analysed with one‐way and two‐way analysis of variance (ANOVA) with Tukey's or Sidak multiple comparisons test and Student's unpaired *t* tests where appropriate. Statistical significance was accepted at *P* < 0.05. Post hoc tests were only conducted when *F* achieved *P* < 0.05 and there was no significant inhomogeneity of variance. For the *in vitro* data IC_50_ values were determined by nonlinear regression using GraphPad Prism 9 (San Diego, California, USA). Sigmoid curves were fitted with a Hill slope of −1.0 for the inhibition curves. The declared group size is the number of independent values and statistical analysis was performed using these independent values.

### 2.5 Materials

mGIPAnt‐1, which is a peptide based on the structure of truncated GIP, was custom synthesized by Peptides & Elephants (Hennigsdorf, Germany). The structure of this peptide is as follows: H‐EGTFISDYSIAMDKIK(C16‐diacid)QQDFVNWLLAQKGKKNDWKHN‐OH. Prior to delivery, the purity of the peptide was determined to be 95.7% and the correct molecular weight was ascertained by mass spectrometry. The peptide was dissolved in 100‐mM NaHCO_3_ containing 0.1% casein (solution from bovine milk—5% in water, Sigma Aldrich, St Louis, USA) prior to use. cDNAs of human and mouse GIPR, as well as mouse GLP‐1 and GLP‐2 receptors, were purchased from OriGene, Rockville, Maryland, USA (SC110906, MC216211, MC216256, MC217338 and MC203290, respectively). GIP (Synthetic mouse GIP(1‐42) was obtained from Caslo peptides, Lyngby, Denmark. Valine pyrrolidide was a gift from Novo Nordisk, Bagsværd, Denmark. Carprofen (Rimadyl) was obtained from Pfizer, New York, NY, USA. Isoflurane was obtained from Baxter, Søborg, Denmark. Ketamine (Ketaminol Vet.) was obtained from MSD Animal Health, Madison, NJ, USA, while xylazine (Rompun Vet.) was obtained from Bayer Animal Health, Leverkusen, Germany. Aprotinin (Trasylol) was obtained from Nordic Group, Hoofddorp, The Netherlands. ^125^I‐monolabelled human GIP(1‐42) (catalogue no. NEX402) was obtained from Perkin Elmer, Skovlunde, Denmark. EDTA‐coated glass capillaries were obtained from Vitrex, Vasekær, Denmark. Eppendorf tubes were obtained from Eppendorf, Hamburg, Germany.

The mouse insulin kit was obtained from Mercodia, Uppsala, Sweden, and the mouse adiponectin ELISA kit was obtained from Crystal Chem, Elk Grove Village, USA. The triglyceride and free glycerol kit was obtained from Sigma‐Aldrich, St. Louis, USA.

### 2.6 Nomenclature of targets and ligands

Key protein targets and ligands in this article are hyperlinked to corresponding entries in the IUPHAR/BPS Guide to PHARMACOLOGY http://www.guidetopharmacology.org and are permanently archived in the Concise Guide to PHARMACOLOGY 2021/22 (Alexander et al., 2021).

## 3 RESULTS

### 3.1 *In vitro* properties *of* mGIPAnt‐1

The antagonistic properties of mGIPAnt‐1 were determined *in vitro* in cAMP accumulation before initiation of the *in vivo* studies in comparison to the naturally occurring GIPR GIP(3‐30)NH_2_. The experiments were done on both the human and mouse GIP receptor to examine the translatability between species. To keep the experiments species specific, mGIPAnt‐1 was compared to mouse GIP(3‐30)NH_2_ (mGIP(3‐30)NH_2_) on the mouse GIP receptor and human GIP(3‐30)NH_2_ (hGIP(3‐30)NH_2_) on the human GIP receptor. We also determined whether mGIPAnt‐1 had any intrinsic activity. Here we observed no activation of the mouse or human GIP receptor and neither did mGIP(3‐30)NH_2_ activate the mouse GIP receptor (Figure 1a,b) as previously shown for hGIP(3‐30)NH_2_ on the human GIP receptor (Gabe et al., 2018; Gasbjerg et al., 2017; Hansen et al., 2016). mGIPAnt‐1 inhibited the mouse GIP receptor with a 3‐fold better potency than mGIP(3‐30)NH_2_ with an IC_50_ of 269 nM compared to 813 nM, respectively (Figure 1a). Focusing on the human GIPR, mGIPAnt‐1 antagonized with an IC_50_ of 11 nM (Figure 1b), which is similar to that of hGIP(3‐30)NH_2_ previously shown to antagonize with an IC_50_ of 12 nM (Hansen et al., 2016).

**FIGURE 2:**
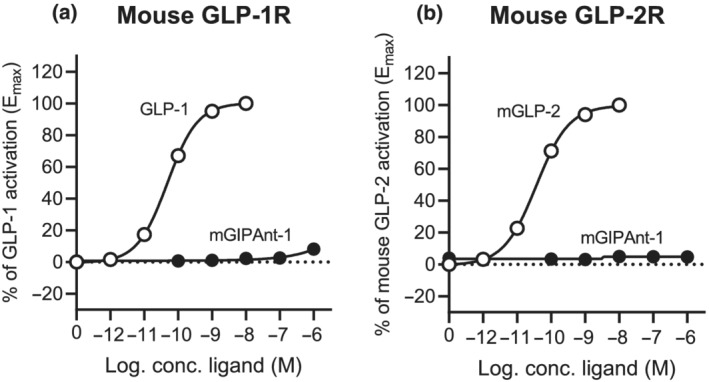
*In vitro* signalling profile of mGIPAnt‐1 on the mouse GLP‐1 and GLP‐2 receptor (R). COS‐7 cells were transiently transfected with (a) mouse GLP1‐R and (b) mouse GLP‐2 R and assayed for cAMP accumulation. Increasing concentrations of either mGIPAnt‐1 or (a) mGLP1, (b) mGLP‐2 were added. Data are shown as mean ± SE from *n* = 5 independent experiments

A second set of experiments showed that mGIPAnt‐1 does not induce activation of the mouse GLP‐1 and GLP‐2 receptors (Figure 2a,b). mGLP‐1 and mGLP‐2 were used as a control and were shown to activate their receptors.

### 3.2 *Pharmacokinetics of* mGIPAnt‐1 *in mice*

**FIGURE 3:**
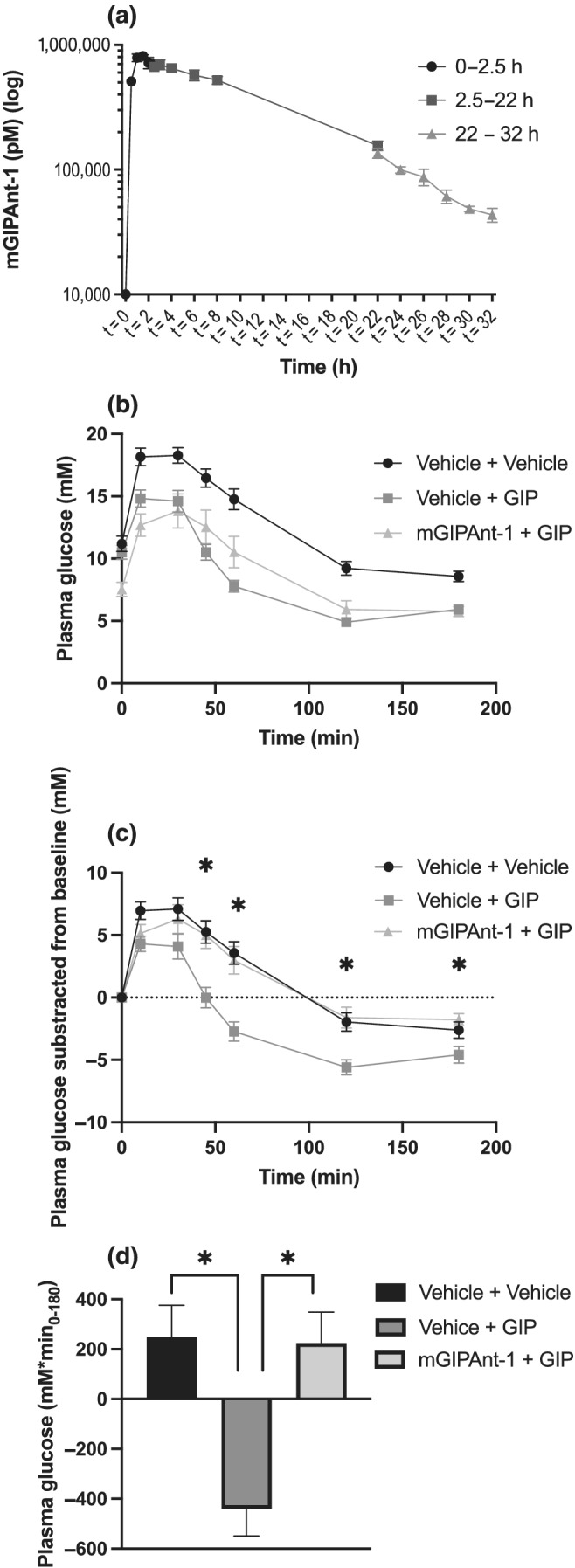
mGIPAnt‐1 pharmacokinetics. (a) mGIPAnt‐1 pharmacokinetics following subcutaneous (s.c.) administration of 6‐nmol mGIPAnt‐1, (b) glucose levels during intraperitoneal glucose tolerance test (IPGTT) following mGIPAnt‐1 (25‐μmol·kg^−1^)/vehicle and gastric inhibitory polypeptide/glucose‐dependent insulinotropic polypeptide (GIP) (25‐nmol·kg^−1^)/vehicle administration, (c) baseline‐subtracted glucose levels during the IPGTT and (d) incremental area under the curve (AUC). Statistical analysis: (b) two‐way analysis of variance (ANOVA) with Tukey Multiple Comparisons test, *P < 0.05, Vehicle + GIP versus *mGIPAnt‐1* + GIP; (c) one‐way ANOVA with Tukey Multiple Comparisons test *P < 0.05. Female C57Bl6/J mice, Vehicle + Vehicle, *n* = 10, Vehicle + GIP, *n* = 12, mGIPAnt‐1 + GIP, *n* = 8. Data are presented as mean ± SE

Figure 3a shows plasma concentrations of mGIPAnt‐1 following s.c. administration (6 nmol per mouse). Plasma concentrations were measured by in‐house radioimmunoassay. To obtain the full elimination curve mice were studied in groups with sampling at different time intervals: 0–2.5, 2.5–22 and 22–32 h. Peak concentration was reached 1.5 h following administration and half‐life was calculated to be approximately 7.2 h.

#### 3.2.1 Acute inhibitory actions of mGIPAnt‐1 on exogenous GIP *in vivo*

We performed an intraperitoneal glucose tolerance test in mice treated with (1) vehicle and vehicle, (2) vehicle and GIP and (3), mGIPAnt‐1 and GIP to determine if mGIPAnt‐1 could inhibit the glucose‐lowering actions of GIP following i.p. glucose administration *in vivo*. Figure 3b shows the glucose curve following glucose administration at *t* = 0 min and Figure 3c shows the glucose curves after subtraction of the baseline value at *t* = 0. Interestingly, animals receiving mGIPAnt‐1 had a significant decrease in fasting blood glucose levels compared to the other two groups. After baseline subtraction (Figure 3c), we see that treatment with GIP alone lowered glucose levels significantly from *t* = 45 min and onwards compared to mice treated with both mGIPAnt‐1 and GIP. The glucose curves of mice treated with vehicle alone did not differ from that of mice treated with both mGIPAnt‐1 and GIP. The incremental area under the curve (iAUC) (Figure 3d) was significantly different for mice treated with vehicle and GIP compared to the double vehicle group and for mice treated both with mGIPAnt‐1 and GIP. We further see that for the animals receiving both mGIPAnt‐1 and GIP, glucose levels do not drop below baseline, whereas at *t* = 180 min, glucose levels did drop significantly below baseline for the animals receiving double vehicle. For the animals receiving GIP alone, glucose levels dropped significantly below baseline at *t* = 120 and *t* = 180 min.

### 3.3 Body weight and body composition of ovariectomised mice following mGIPAnt‐1 treatment

**FIGURE 4:**
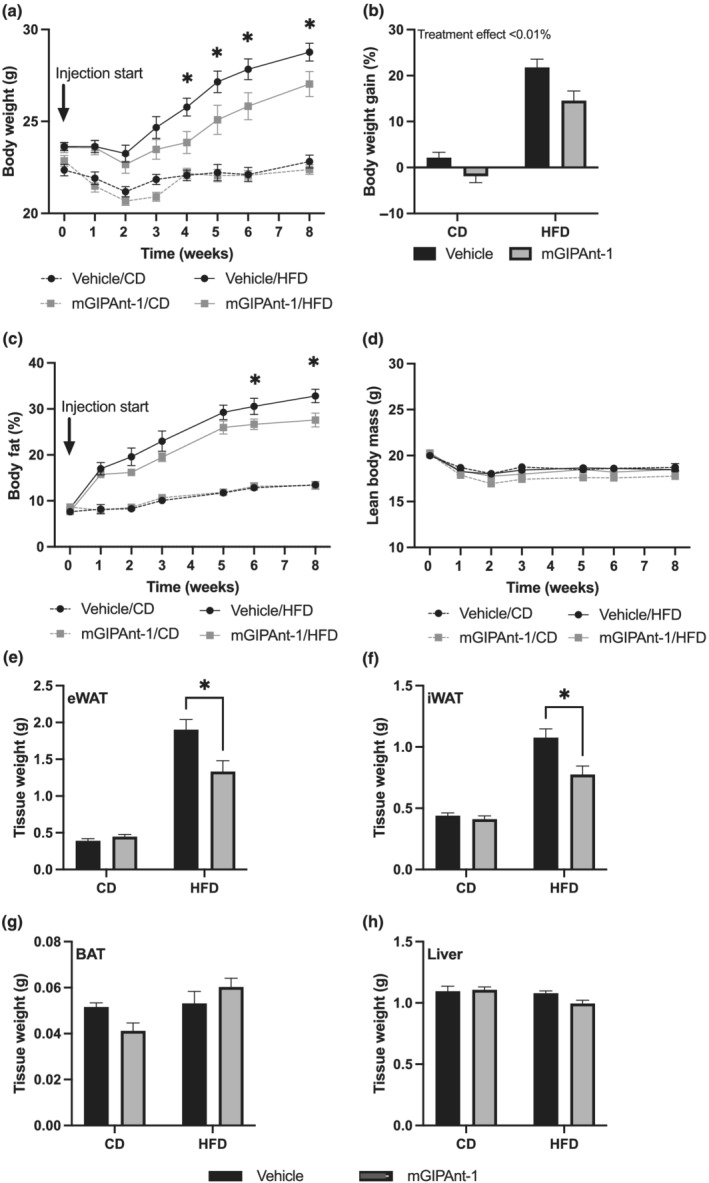
Body weight and composition following high fat diet (HFD) and mGIPAnt‐1 treatment. (a) Body weight, (b) body weight gain (%), (c) body fat (%), (d) lean body weight and terminal tissue weight, (e) epidydimal white adipose tissue (eWAT), (f) inguinal white adipose tissue (iWAT), (g) brown adipose tissue (BAT) and (H) liver. Statistical analysis: (a, c) two‐way analysis of variance (ANOVA) with Tukey Multiple Comparisons test *P < 0.05, Vehicle/HFD versus mGIPAnt‐1/HFD; (b, d–g) two‐way ANOVA with Sidak Multiple Comparison Test; *P < 0.05. Female ovariectomised C57Bl6/J mice, Vehicle/Conrol Diet (CD), *n* = 12; mGIPAnt‐1/CD, *n* = 12; Vehicle/HFD, *n* = 11; mGIPAnt‐1/HFD, *n* = 12 (*n* = 6 for C). Data are presented as mean ± SE

A week following ovariectomy, mice were placed on either control diet or HFD and daily treatment with mGIPAnt‐1 or vehicle (control) commenced. Figure 4a shows that from 4 weeks onwards, body weight in the control, HFD group was significantly higher compared to the mGIPAnt‐1/HFD group, whereas absolute body weight was not affected by mGIPAnt‐1 treatment in animals fed control diet. We observed an overall treatment effect of mGIPAnt‐1, reducing total body weight gain over the course of the experiment (Figure 4a). As expected, we also observed an overall diet effect (Figure 4b). Final lean body mass was not affected by mGIPAnt‐1 treatment (Figure 4d), whereas at 6 and 8 weeks, Control/HFD animals significantly increased body fat percentage compared to mGIPAnt‐1/HFD animals (Figure 4c). Weight of both epididymal white adipose tissue (eWAT) and inguinal white adipose tissue (iWAT) were reduced in mGIPAnt‐1/HFD mice compared to Vehicle/HFD mice, whereas mGIPAnt‐1 treatment did not affect eWAT and iWAT weight in control diet‐fed mice (Figure 4e,f). BAT and liver weight was not affected by mGIPAnt‐1 treatment (Figure 4g,h).

### 3.4 *Glycaemic control following* mGIPAnt‐1 *treatment*

**FIGURE 5:**
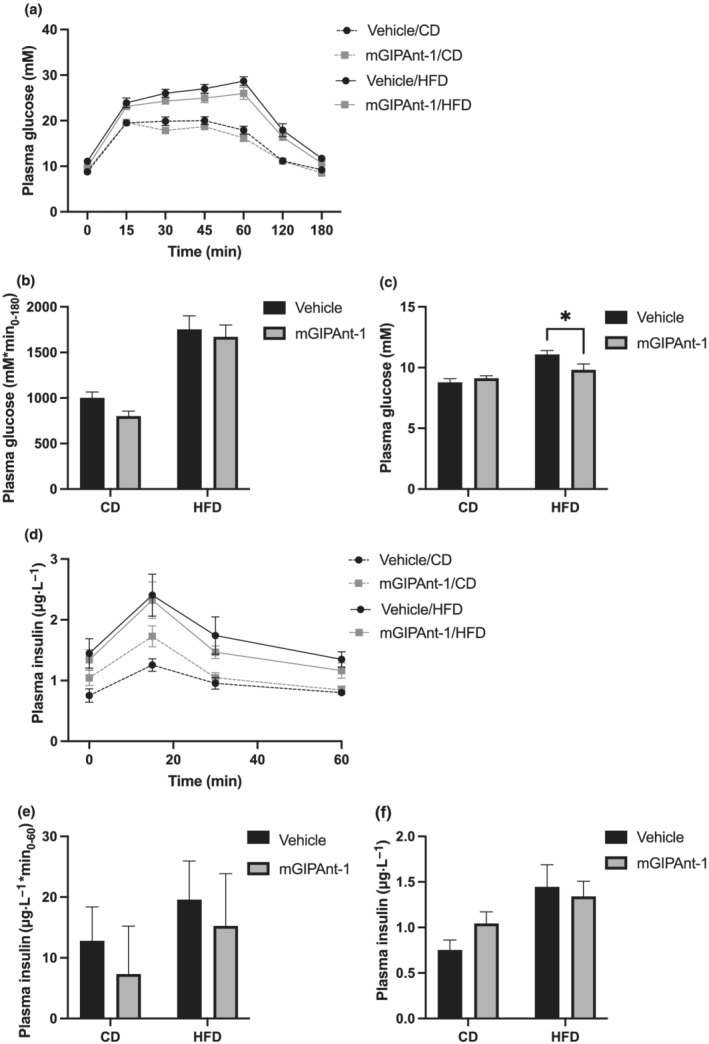
Oral glucose tolerance test (OGTT). (a) Glucose and (d) insulin levels, and (b) glucose and (e) incremental area under the curve following 2‐g·kg^−1^ oral glucose, (c) fasting glucose and (f) insulin levels. Statistical analysis: (a, d) two‐way analysis of variance (ANOVA) with Tukey Multiple Comparisons test; (b, c, e and f) two‐way ANOVA with Sidak Multiple Comparisons test, *P <0.05. Female ovariectomised C57Bl6/J mice, Vehicle/Control Diet (CD), *n* = 12; mGIPAnt‐1/CD, *n* = 12; Vehicle/high fat diet (HFD, *n* = 11; mGIPAnt‐1/HFD, *n* = 12. Data are presented as mean ± SE

Whereas HFD feeding increased glucose and insulin levels during an oral glucose tolerance test, mGIPAnt‐1 treatment for 7 weeks did not affect glucose or insulin curves nor the respective iAUCs (Figures 5a,b and 5d,e). However, mGIPAnt‐1/HFD mice did have significantly decreased fasting glucose levels compared to Vehicle/HFD mice (Figure 5c). Fasting insulin levels were not affected by mGIPAnt‐1 treatment, although HFD did significantly increase fasting insulin levels (Figure 5f).

### 3.5 Terminal plasma levels of adiponectin, triglycerides and glycerol

**FIGURE 6:**
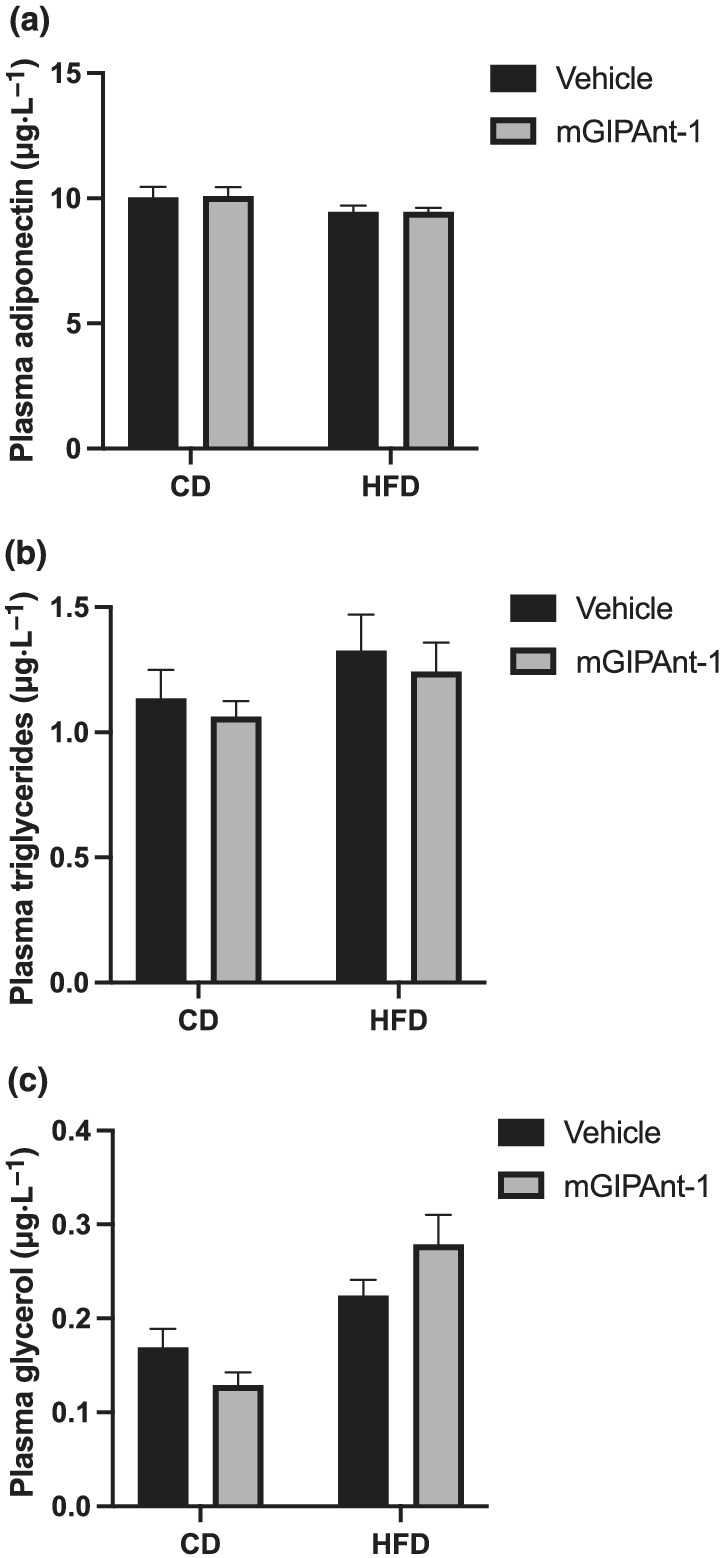
Biochemical analysis. Terminal plasma levels of (a) adiponectin, (b) triglyceride and (c) glycerol. Statistical analysis: two‐way analysis of variance (ANOVA) with Sidak Multiple Comparisons test. Female ovariectomised C57Bl6/J mice, Vehicle/Control Diet (CD), *n* = 12; mGIPAnt‐1/CD, *n* = 12; Vehicle/high fat diet (HFD, *n* = 11; mGIPAnt‐1/HFD, *n* = 12. Data are presented as mean ± SE

mGIPAnt‐1 treatment did not significantly affect plasma adiponectin or triglyceride levels or glycerol levels (Figure 6). mGIPAnt‐1/HFD mice showed a trend towards increased glycerol levels when compared to Vehicle/HFD mice, but this did not reach significance.

## 4 DISCUSSION AND CONCLUSIONS

Antagonism of the GIP receptor has received much attention as a potential target for obesity therapy, strongly supported by studies showing that deletion of the GIP receptor in mice resulted in resistance to diet‐induced weight gain (Boer, Keenan, et al., 2021; Hansotia et al., 2007; Miyawaki et al., 2002; Naitoh et al., 2008). In this study, we characterized the *in vitro* and *in vivo* pharmacological properties of a novel GIPR antagonist, mGIPAnt‐1. Secondly, we show that sub‐chronic treatment with mGIPAnt‐1 results in a reduction of body weight gain in ovariectomised mice.

mGIPAnt‐1 is a truncation of the GIP sequence, which was acylated to enhance *in vivo* half‐life. A similar strategy has been applied to prolong the activity of other GIP antagonists. (Pro^3^)GIP, in which Glu^3^ is substituted with Pro^3^, was demonstrated to be resistant to enzymatic degradation (Gault et al., 2002). This compound was acylated (e.g. Pro^3^ GIPLys^16^PAL) or mPEGylated (Pro^3^GIP[mPEG]) to enhance bioactivity of the antagonist, although this was only achieved after mPEGylation (Gault et al., 2007; McClean et al., 2008). (Pro^3^)GIP and its variants showed promising effects in murine models; treatment induced a decrease in body weight gain in HFD‐fed mice, improved glucose tolerance and enhanced insulin sensitivity (McClean et al., 2008). It was, however, later discovered that Pro^3^GIP is not a full antagonist, but rather a partial agonist of the rodent GIP receptor and a full *agonist* of the human GIP receptor (Sparre‐Ulrich et al., 2016). This finding also highlighted the importance of interspecies differences in the GIP system (Sparre‐Ulrich et al., 2016). While human GIP(1‐30)NH_2_ is a full agonist at the human GIP receptor, human GIP(3‐30)NH_2_ is a very selective and competitive antagonist and has been used as a tool to study GIP physiology in humans (Gasbjerg et al., 2017; Gasbjerg et al., 2021) and a species‐specific variant was used in rodent studies (Perry et al., 2019). However, due to its short half‐life, GIP(3–30)NH_2_ has so far only been used in acute studies in humans (Lynggaard et al., 2020). GIP(1‐39)NH_2_ is a particularly potent agonist of the murine GIP receptor (Xie et al., 2004) and based on this observation, a screening of peptides based on the GIP(3‐39) backbone involving relevant truncations was conducted on the mouse GIP receptor and mGIPAnt‐1 was selected as the most potent antagonist.

In this study, we first confirmed *in vitro* that mGIPAnt‐1 was capable of inhibiting cAMP accumulation induced by bio‐active, species‐specific GIP(1‐42) at both the human and mouse GIP receptor, and that it did so to a similar degree as mGIP(3‐30)NH_2_ on the mouse GIP receptor. Furthermore, our pharmacokinetic *in vivo* studies showed that following s.c. administration, peak values were reached after 1.5 h and half‐life of the compound was approximately 7.2 h. *In vivo* GIP receptor antagonism by mGIPAnt‐1 at a dose 1000 times that of mGIP(1‐42) was confirmed with an intraperitoneal glucose tolerance test in which we observed that the antagonist inhibited the net decrease of plasma glucose levels induced by GIP injection in control experiments. Interestingly, animals receiving mGIPAnt‐1 had a decrease in baseline blood glucose levels. As we learned from our *in vitro* data, mGIPAnt‐1 did not activate the GIP receptor or the GLP‐1 receptor. We are therefore uncertain why injection of mGIPAnt‐1 in lean mice acutely resulted in a decrease in fasting blood glucose levels.

We next utilized an ovariectomised mouse model to study the effects of sub‐chronic treatment of mGIPAnt‐1 on body weight and composition and glucose regulation. As mice consume the majority of their food during the dark‐phase (Kurokawa et al., 2000), we decided from our pharmacokinetic data that a once‐daily injection, 1.5 h before the dark phase would be sufficient to induce antagonism of endogenously secreted GIP following food ingestion. There were several reasons for us to use the ovariectomised mice. Rodent ovariectomy models mimic menopause and the drop of plasma oestrogen levels following removal of the ovaries results in increased fat mass and body weight gain (Kalu & Chen, 1999; McElroy & Wade, 1987). Due to their decline of oestrogen levels and reduced energy expenditure, postmenopausal women are at higher risk for the development of obesity and insulin resistance (Isken et al., 2008). Furthermore, postmenopausal women have increased plasma GIP levels (Ranganath et al., 1998), which are reduced by oestrogen replacement therapy (Sztefko et al., 2005). GIP receptor KO in ovariectomised mice causes, as for male mice, resistance to diet‐induced weight gain (Isken et al., 2008; Shimazu‐Kuwahara et al., 2019). Obesity treatment based on GIP receptor antagonism could therefore be of relevance to this part of the population. The use of females in our studies was also a practical consideration; male mice are more aggressive, which may result in injuries. The stress related to this may increase data variability, thereby requiring larger group sizes to increase statistical power. When wounds inflicted by fighting are severe, mice must be killed for ethical considerations, negatively impacting group size. Females, on the other hand, are less aggressive and easier to house together (Lidster et al., 2019).

Daily treatment with mGIPAnt‐1 significantly reduced body weight in HFD‐fed ovariectomised mice after 4 weeks of treatment and when assessing overall body weight gain over the 8‐week treatment period, we observed an overall reducing effect of mGIPAnt‐1 treatment on body weight gain. This increase was due to a change in fat mass, rather than due to a change in lean body mass. This was further confirmed by decreased tissue weights of both eWAT and iWAT.

Whereas mGIPAnt‐1 acutely inhibited the glucose‐lowering and insulin‐releasing actions of exogenously administered GIP, no effects of sub‐chronic mGIPAnt‐1 treatment on glucose and insulin curves during an oral glucose tolerance test were observed. We did, however, observe reduced fasting levels in HFD‐fed female mice treated with the antagonist compared to vehicle‐treated animals on the same diet. These results are similar to those observed in mice treated with a GIP receptor antibody, which also exhibited lower fasting glucose levels but no change in glucose curves during an oral glucose tolerance test (Killion et al., 2018). However, those mice also exhibited lower fasting insulin levels, whereas fasting insulin levels were unchanged in the current study.

Now that we have established that mGIPAnt‐1 functions as an antagonist of the murine GIP receptor, has a half‐life that allows for once‐daily dosing and induces a reduction in weight gain in mice, it is of great interest to further explore the mechanism behind these anti‐obesogenic effects. Due to technical difficulties, we were, unfortunately, unable to obtain food‐intake data. Previous studies using GIP antagonists have shown varying effects with respect to food intake. Treatment of diet‐induced obese mice with an antagonistic GIP receptor antibody reduced food consumption (Killion et al., 2018), whereas the small‐molecule compound SKL‐14959 suppressed weight gain without affecting food consumption (Nakamura et al., 2018). Whereas studies utilizing mouse models of GIP receptor KO have consistently shown that GIP receptor KO results in resistance to the development of obesity, conflicting data have been reported on food intake, with one study showing decreased food intake in the GIP receptor KO mice (Hansotia et al., 2007) and others showing no difference between GIP receptor KO and WT groups (Boer, Keenan, et al., 2021; Miyawaki et al., 2002; Naitoh et al., 2008). Double incretin receptor knockout mice likewise are resistant to diet‐induced obesity, although this is paired with an increase in food intake compared to WT littermates, when corrected for body weight (Hansotia et al., 2007). Further studies are required to assess how treatment with mGIPAnt‐1 could affect food intake in mice. In a recent study, we showed that the resistance to diet‐induced weight gain commonly shown in GIP receptor KO mice can be at least in part be explained by enhanced energy expenditure and activity levels, as well as increased lipolysis in iWAT and eWAT (Boer, Keenan, et al., 2021). Interestingly, we found that GIP receptor KO had increased postprandial lipid storage in iWAT. It would be highly interesting to investigate whether the resistance to weight gain that we observed following GIP antagonist treatment in this study is due to similar mechanisms. It was recently reported the GIP receptor expression in white adipose tissue is mainly localized to non‐adipocyte cell types rather than adipocytes. This does, however, not rule out a role for GIP receptor signalling in adipose tissue (Campbell et al., 2022).

Recent studies have indicated that GIP may have a central action on food intake and energy expenditure, which are both centrally regulated. Central infusion of an antagonistic GIP receptor antibody reduced food intake and lowered body weight in diet‐induced obese mice (Kaneko et al., 2019). It was proposed that these actions were achieved through inhibition of GIP‐induced leptin resistance. Mice with central GIP receptor KO were likewise protected from diet‐induced weight gain and showed reduced food intake (Zhang et al., 2021). However, central infusion of acyl‐GIP (a long‐acting GIP) and chemogenetic activation of GIP receptor expressing cells in the central nervous system also suppressed food intake in diet‐induced obese mice and chow‐fed mice, respectively (Adriaenssens et al., 2019; Zhang et al., 2021). These contradicting studies highlight the importance of studying how mGIPAnt‐1 could affect central GIP actions. Finally, it would be highly interesting to investigate whether effects on weight gain as observed in the ovariectomised mouse model may also be found in other models of dietary obesity. Further research is therefore required regarding the effects of mGIPAnt‐1 in other models of obesity.

Killion et al. (2018) showed that in both obese mice and non‐human primates, treatment with a combination of an antagonistic GIP receptor antibody and the GLP‐1 receptor agonist dulaglutide resulted in greater reduction of weight gain compared to either treatment alone. Paradoxically, however, in both clinical and preclinical trials, combining a GLP‐1 agonist with a GIP agonist in a compound such as tirzepatide, likewise resulted in a more positive treatment outcome compared to treatment with the GLP‐1 agonist alone as shown by the SURPASS trials (Dahl et al., 2021; Frias et al., 2018; Ludvik et al., 2021; Nørregaard et al., 2018; Rosenstock et al., 2021). Even though a definite function of the GIP compound in this molecule on food intake and body weight has not yet been uncovered, it has been suggested that there may be a synergy of the effects of both GIP and GLP‐1 on central satiety and weight loss (Bailey, 2021). Furthermore, preclinical studies have reported that the GIP component in tirzepatide treatment in diet‐induced obese mice significantly improves insulin sensitivity in treated mice (Samms et al., 2021). With the development of mGIPAnt‐1, we have created a novel tool compound to further study the effects of GIP receptor antagonism in the context of obesity.

In conclusion, we demonstrate that the tool compound developed to antagonize the GIP receptor in mice, mGIPAnt‐1, is capable of acutely inhibiting GIP's glucoregulatory and insulinotropic effects *in vivo* and induces resistance to HFD‐induced weight gain in ovariectomised mice.

## CONFLICT OF INTEREST

MBNG, AHSU, JJH and MMR are co‐founders of Antag Therapeutics ApS. The remaining authors declare that they do not have a conflict of interest.

## AUTHOR CONTRIBUTIONS

GAB, AHSU, BH, JJH and MMR designed the study. GAB, JEH, MNG and JAW performed the experiments and analysed data. GAB wrote the manuscript. JEH, MBNG, JAW, AHSU, BH, JJH and MMR critically reviewed and edited the manuscript. All authors agreed to its publication.

## DECLARATION OF TRANSPARENCY AND SCIENTIFIC RIGOUR

This Declaration acknowledges that this paper adheres to the principles for transparent reporting and scientific rigour of preclinical research as stated in the *BJP* guidelines for Design and Analysis and Animal Experimentation, and as recommended by funding agencies, publishers and other organisations engaged with supporting research.

## Data Availability

The data that support the findings of this study are available from the corresponding author upon reasonable request. Some data may not be made available because of privacy or ethical restrictions.
